# Safety of systemic anti-cancer treatment in oncology patients with non-severe COVID-19: a cohort study

**DOI:** 10.1186/s12885-021-08349-8

**Published:** 2021-05-20

**Authors:** C. van Marcke, N. Honoré, A. van der Elst, S. Beyaert, F. Derouane, C. Dumont, F. Aboubakar Nana, J. F. Baurain, I. Borbath, P. Collard, F. Cornélis, A. De Cuyper, F. P. Duhoux, B. Filleul, R. Galot, M. Gizzi, F. Mazzeo, T. Pieters, E. Seront, I. Sinapi, M. Van den Eynde, N. Whenham, J. C. Yombi, A. Scohy, A. van Maanen, J. P. Machiels

**Affiliations:** 1 Department of Medical Oncology, Institut Roi Albert II, Cliniques universitaires Saint-Luc, Avenue Hippocrate 10, 1200 Brussels, Belgium; 2 Institute for Experimental and Clinical Research (IREC, pôle MIRO), Université catholique de Louvain (UCLouvain), Avenue Hippocrate 10, 1200 Brussels, Belgium; 3 Department of Pneumology, Institut Roi Albert II, Cliniques universitaires Saint-Luc, Brussels, Belgium; 4 Institute for Experimental and Clinical Research (IREC, pôle PNEU), Université catholique de Louvain (UCLouvain), Brussels, Belgium; 5 Department of Hepatogastroenterology, Institut Roi Albert II, Cliniques universitaires Saint-Luc, Brussels, Belgium; 6 Department of Medical Oncology, Hôpital de Jolimont, Haine-Saint-Paul, Belgium; 7 Department of Medical Oncology, Grand Hôpital de Charleroi (GHdC), Charleroi, Belgium; 8 Department of Medical Oncology, Clinique Saint-Pierre, Ottignies, Belgium; 9 Department of General Internal Medicine, Cliniques universitaires Saint-Luc, Brussels, Belgium; 10 Department of Microbiology, Cliniques universitaires Saint-Luc, Brussels, Belgium; 11 Statistics unit, Institut Roi Albert II, Cliniques universitaires Saint-Luc, Brussels, Belgium

**Keywords:** Systemic anti-cancer treatment, Non-severe COVID-19, Ambulatory, Safety

## Abstract

**Background:**

The viral pandemic coronavirus disease 2019 (COVID-19) has disrupted cancer patient management around the world. Most reported data relate to incidence, risk factors, and outcome of severe COVID-19. The safety of systemic anti-cancer therapy in oncology patients with non-severe COVID-19 is an important matter in daily practice.

**Methods:**

ONCOSARS-1 was a single-center, academic observational study. Adult patients with solid tumors treated in the oncology day unit with systemic anti-cancer therapy during the initial phase of the COVID-19 pandemic in Belgium were prospectively included. All patients (*n* = 363) underwent severe acute respiratory syndrome coronavirus-2 (SARS-CoV-2) serological testing after the first peak of the pandemic in Belgium. Additionally, 141 of these patients also had a SARS-CoV-2 RT-PCR test during the pandemic. The main objective was to retrospectively determine the safety of systemic cancer treatment, measured by the rate of adverse events according to the Common Terminology Criteria for Adverse Events, in SARS-CoV-2-positive patients compared with SARS-CoV-2-negative patients.

**Results:**

Twenty-two (6%) of the 363 eligible patients were positive for SARS-CoV-2 by RT-PCR and/or serology. Of these, three required transient oxygen supplementation, but none required admission to the intensive care unit. Hematotoxicity was the only adverse event more frequently observed in SARS-CoV-2 -positive patients than in SARS-CoV-2-negative patients: 73% vs 35% (*P* < 0.001). This association remained significant (odds ratio (OR) 4.1, *P* = 0.009) even after adjusting for performance status and type of systemic treatment. Hematological adverse events led to more treatment delays for the SARS-CoV-2-positive group: 55% vs 20% (*P* < 0.001). Median duration of treatment interruption was similar between the two groups: 14 and 11 days, respectively. Febrile neutropenia, infections unrelated to COVID-19, and bleeding events occurred at a low rate in the SARS-CoV-2-positive patients.

**Conclusion:**

Systemic anti-cancer therapy appeared safe in ambulatory oncology patients treated during the COVID-19 pandemic. There were, however, more treatment delays in the SARS-CoV-2-positive population, mainly due to a higher rate of hematological adverse events.

**Supplementary Information:**

The online version contains supplementary material available at 10.1186/s12885-021-08349-8.

## Background

The viral pandemic coronavirus disease 2019 (COVID-19), caused by the severe acute respiratory syndrome coronavirus-2 (SARS-CoV-2), can present broadly, from asymptomatic infection through symptoms of mild general upper respiratory infection, life-threatening acute respiratory distress syndrome, and death [1].

Acute infection is diagnosed by reverse transcription polymerase chain reaction (RT-PCR) allowing the detection of viral RNA from a nasopharyngeal swab. Unfortunately, logistic, financial, and sometimes legal issues precluded during the first wave of the pandemic the vast majority of caregivers around the world, including us, to test patients before each hospital admission, treatment, or diagnostic procedure [2]. Early in the pandemic, specific infection prevention measures and recommendations were implemented in hospitals. These aimed to identify infected patients before they entered the oncology department, protect patients and staff, and maintain treatment quality [3–5]. Drastic reductions in cancer diagnosis in the latest months is another dramatic consequence of the pandemic [6].

The majority of scientific data regarding COVID-19 infection in cancer patients relate to the severe form of the disease and focus mainly on incidence, risk factors and outcomes [7–9]. Several meta-analyses suggest that cancer patients are at higher risk of developing the disease and having a severe form, but also present higher mortality rates [10–12]. Furthermore, large collaborative registries suggest that COVID-19 positive lung cancer patients are at highest risk of dismall prognosis [13, 14].

However, around 80% of COVID-19-positive patients in the general population have only mild symptoms of the disease, and an undefined number are asymptomatic transmitters [1]. It is of utmost importance to collect more data regarding the safety of systemic anti-cancer treatments in cancer patients with a non-severe form of COVID-19 [15, 16]. Indeed, in the presence of mild symptoms, COVID-19 infection can remain undetected due to the absence or unavailability of repeatable rapid screening methods. Access to SARS-CoV-2 RT-PCR testing was limited in Belgium during the initial pandemic due to logistics; its availability focused mainly on patients with severe COVID-19 that required hospitalization. The Belgian authorities authorized serological testing to detect post-infection antibodies in the population on May 20, 2020. As we did not screen asymptomatic patients or those with only mild symptoms during the pandemic, we suspected that some of our patients with non-severe SARS-CoV-2 infection have received systemic anti-cancer treatment. We therefore undertook a prospective study between June 12 and July 13, 2020 and offered serological testing to each cancer patient undergoing systemic treatment in our cancer day unit. Our main objective was to retrospectively report the complication rates of systemic treatment in cancer patients with non-severe COVID-19 infection, identified by SARS-CoV-2 RT-PCR or serology, in comparison to SARS-CoV-2-negative patients. We also aimed to identify the discriminant symptoms and clinical/radiological factors associated with SARS-CoV-2 positivity, and to estimate the seroprevalence of antibodies against SARS-CoV-2 in this population.

## Methods

### Study design, inclusion criteria, study objectives and endpoints

ONCOSARS-1 was a single center, academic observational study of a cohort prospectively collected at Institut Roi Albert II, Cliniques universitaires Saint-Luc (CUSL), a tertiary cancer center and the largest in Brussels, Belgium. The inclusion criteria were: (i) patients older than 18 years, (ii) with a diagnosis of active solid cancer, (iii) having received systemic anti-cancer therapy between February 15 and May 31, 2020, (iv) admitted to the oncology day unit between June 12 and July 13, 2020, (v) who agreed to have a SARS-CoV-2 serological test and (vi) who signed an informed consent. There were no exclusion criteria. Hematological cancer patients are treated in a separate outpatient unit and did not take part in this study.

In this selected group of patients, our main objective was to retrospectively investigate the safety of systemic anti-cancer treatment administered to SARS-CoV-2-positive patients in a day unit setting. By doing so, we also aimed to identify the discriminant symptoms and clinical or radiological factors associated with SARS-CoV-2 positivity, and to estimate the seroprevalence of SARS-CoV-2 in this population. The primary endpoint was the rate of adverse events in SARS-CoV-2-positive patients compared to SARS-CoV-2-negative patients according to the Common Terminology Criteria for Adverse Events (CTCAE) version 5.0.

SARS-CoV-2-positive patients were defined as patients in whom SARS-CoV-2 RNA was detected by RT-PCR in a nasopharyngeal swab between February 15 and July 13, 2020, or as patients with a positive SARS-CoV-2 serological test between June 12 and July 13, 2020. The study was approved by our Ethics Committee (2020/18MAI/278) on June 8, 2020, and all patients signed an informed consent.

In parallel to this study, all cancer healthcare workers had the opportunity to have a SARS-CoV-2 serological test at the same timepoint following informed consent.

### SARS-CoV-2 serological test and RT-PCR

Qualitative detection of SARS-CoV-2 total antibodies in human serum was performed on the Cobas e602 using Elecsys anti-SARS-CoV-2 electrochemiluminescent immunoassay (ECLIA) (Roche Diagnostics, Mannheim, Germany). This assay uses a recombinant nucleocapsid antigen for the detection of anti-SARS-CoV-2 antibodies. According to the manufacturer’s insert, a cutoff index of ≥1.0 indicates a positive result [17].

SARS-CoV-2 RNA detection in nasopharyngeal swabs relies on the genesig® Real-Time RT-PCR assay (Primerdesign Ltd., Chandler’s Ford, United Kingdom). This assay, performed on RNA extracts, allows the detection of viral RNA by targeting the RNA-dependent RNA polymerase (RdRp) gene. The amplification was performed on a LightCycle 480 instrument (Roche Diagnostics, Mannheim, Germany) according to the manufacturer’s recommendations. A test with a cycle threshold under 40 was considered positive.

### Data extraction

Data were systematically collected for all patients admitted to our oncology unit. At the beginning of the pandemic, the pre-defined data template was adapted to highlight COVID-19 symptoms (Supplementary appendix 1). Clinical data regarding patient demographics, comorbidities, cancer type and stage, oncological treatment, adverse events (graded according to CTCAE v5.0), COVID-19 related symptoms (fever, cough, anosmia, dyspnea and rhinitis), thoracic imaging and SARS-CoV-2 testing (RT-PCR and serology) were collected and managed using REDCap (Research Electronic Data Capture) tools hosted at CUSL [18, 19]. Data were collected during the initial pandemic outbreak in Belgium (February 15 – May 31, 2020).

### Statistical analysis

The distribution of clinical characteristics, thoracic imaging findings and outcomes between SARS-CoV-2-positive and -negative patients (as detected through RT-PCR or serology) was compared using the Fisher’s exact or Mann-Whitney test, as appropriate. Bonferroni correction was applied for multi-testing. The frequency of treatment-related adverse events was compared using Fisher’s exact test. Univariate and multivariate logistic regression modelling was used to identify risk factors associated with (i) adverse events significantly more frequent in SARS-CoV-2- positive patients and (ii) SARS-CoV-2 seroconversion. The threshold to include candidate variables in the multivariate model was set at 10%, and a backward stepwise selection was used to obtain the optimal model. In all analyses, *p*-values were 2-tailed, and *P*-values < 0.05 were considered statistically significant. Data analyses were performed using SAS software (version 9.4, SAS Institute Inc., NC, USA).

## Results

### Patient characteristics and incidence of SARS-CoV-2 detection

**Fig. 1:**
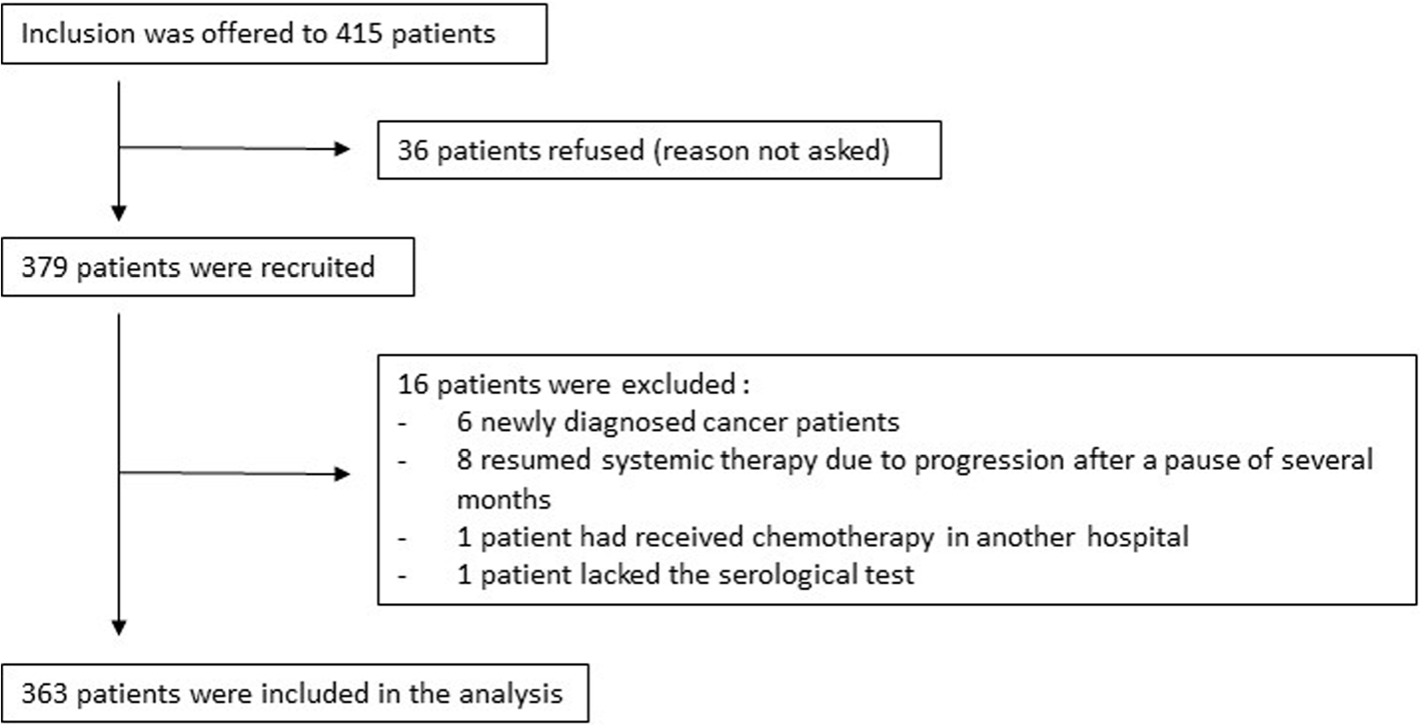
Flowchart showing patient enrollment and exclusion

Of the 415 patients admitted to our oncology day unit between June 12 and July 13, 2020, 379 (91%) signed the informed consent and 363 (87%) met our inclusion criteria (Fig. 1). Table 1 outlines the main patient characteristics. All eligible patients meeting the inclusion criteria (*n* = 363) had SARS-CoV-2 serology performed. Among these, 141 (38.8%) underwent RT-PCR during the COVID-19 pandemic: 22 (16%) had symptoms suggestive of a SARS-CoV-2 infection that triggered the test and 119 (84%) were tested as part of a systematic screening plan. This plan, introduced by CUSL, from April 1, 2020, mandated that all patients planned for a medical procedure or overnight stay must undergo systematic screening with SARS-CoV-2 RT-PCR. However, this systematic screening was not implemented in the outpatient oncology day unit, mainly due to logistical reasons.

**Table 1:** Patients’ characteristics

	All (*N* = 363)	SARS-CoV-2 positive * (*N* = 22)	SARS-CoV-2 negative ^**$**^ (*N* = 341)	***P***
SARS-CoV-2 RT-PCR test performed	141 (39%)	19 (86%)	122 (36%)	NA
Positive	14 (4%)	14 (64%)	0	
Negative	127 (35%)	5 (23%)	122 (36%)	
SARS-CoV-2 serological test performed	363 (100%)	22 (100%)	341 (100%)	NA
Positive	17 (5%)	17 (77%)	0	
Negative	346 (95%)	5 (23%)	341 (100%)	
Age				
Median (IQR)	63 (54–70)	56 (42–68)	63 (56–71)	0.124
≥ 65 years old	159 (44%)	8 (36%)	151 (44%)	0.514
Sex				0.509
Male	167 (46%)	12 (55%)	155 (46%)	
Female	196 (54%)	10 (45%)	186 (55%)	
Comorbidities	195 (54%)	12 (55%)	183 (54%)	0.999
Arterial hypertension	125 (34%)	7 (32%)	118 (35%)	0.999
Diabetes	43 (12%)	2 (9%)	41 (12%)	0.999
Cirrhosis	6 (2%)	0	6 (2%)	0.999
CKD	31 (9%)	3 (14%)	28 (8%)	0.419
COPD	19 (5%)	1 (5%)	18 (5%)	0.999
Heart disease	43 (12%)	4 (18%)	39 (11%)	0.314
Auto-immune disease	23 (6%)	2 (9%)	21 (6%)	0.641
Cancer type				**0.005** ^**γ**^
Breast/gynecological	111 (31%)	6 (27%)	105 (31%)	0.999
Genito-urinary	27 (7%)	1 (5%)	26 (8%)	0.999
Lung	58 (16%)	4 (18%)	54 (16%)	0.999
Digestive tract	93 (26%)	7 (32%)	86 (25%)	0.999
Skin	30 (8%)	0	30 (9%)	0.190
Head and neck	18 (5%)	0	18 (5%)	0.999
Sarcoma	12 (3%)	1 (5%)	11 (3%)	0.999
Other ^α^	14 (4%)	3 (14%)	11 (3%)	0.364
Cancer stage				
Loco-regional	121 (33%)	10 (46%)	111 (33%)	0.245
Metastatic	242 (67%)	12 (55%)	230 (68%)	0.691
Lung	88 (24%)	5 (23%)	83 (24%)	
Other visceral	86 (24%)	5 (23%)	81 (24%)	
Non-visceral	68 (19%)	2 (9%)	66 (19%)	
ECOG Performance status				0.707
- 0–1	351 (97%)	22 (100%)	329 (96%)	
- 2–3	12 (3%)	0	12 (4%)	
Cancer treatment				**0.029** ^**γ**^
Chemotherapy alone	179 (49%)	15 (68%)	164 (48%)	0.320
Immunotherapy alone	104 (29%)	1 (5%)	103 (30%)	**0.028**
Chemotherapy + immunotherapy	22 (6%)	2 (9%)	20 (6%)	0.999
Other ^β^	58 (16%)	4 (18%)	54 (16%)	0.999
Line of treatment if metastatic (mean ± SD)	2.0 ± 1.3	1.8 ± 1.1	2.0 ± 1.4	
Other factors				
Smoker	166 (46%)	9 (41%)	157 (46%)	0.666
Thoracic radiotherapy < 6 months ago	41 (11%)	7 (32%)	34 (10%)	**0.007**
Heavy surgery < 6 months ago	44 (12%)	3 (14%)	41 (12%)	0.291
Symptoms suggestive of COVID-19				
Any	150 (41%)	12 (55%)	138 (41%)	0.194
Fever, cough or anosmia	54 (15%)	10 (46%)	44 (13%)	**< 0.001**
Fever	18 (5%)	5 (23%)	13 (4%)	0.273
Cough	43 (12%)	6 (27%)	37 (11%)	0.185
Anosmia	5 (1%)	5 (23%)	0	**< 0.001**
Rhinitis	30 (8%)	4 (18%)	26 (8%)	0.707
Dyspnea	125 (34%)	10 (46%)	115 (34%)	0.999
Thoracic imaging				
Performed	280 (77%)	14 (64%)	266 (78%)	0.123
COVID-19 suspected	31 (9%)	8 (37%)	23 (7%)	**< 0.001**

* defined as patients with SARS-CoV-2 detected by serology or RT-PCR

^$^Defined as patients with a negative serology test and either a negative RT-PCR or no RT-PCR performed

^α^ Eye melanoma, chordoma, multiple primary tumor, thymic carcinoma, brain tumors

^β^ Targeted therapy or antibody drug conjugate, approved or as part of a clinical study

^**γ**^ Overall Fisher p-value. Subgroups *p*-values are derived from the Fisher test with Bonferroni correction

*SARS-CoV-2* severe acute respiratory syndrome coronavirus-2, *ECOG* Eastern Cooperative Oncology Group, *COPD* chronic obstructive pulmonary disease, *CKD* chronic kidney disease, *RT-PCR* reverse-transcriptase polymerase chain reaction, *SD* standard deviation. COVID-19: The viral pandemic coronavirus disease 2019; *NA* non-applicable

According to RT-PCR and/or serological test, 22 (6%) of the 363 eligible patients had been exposed to SARS-CoV-2. One hundred forty-one patients were tested by RT-PCR, 14 (10%) were positive. For RT-PCR, the positivity rate varied largely between symptomatic and systematically screened patients: 9 out of 22 (41%) versus 5 out of 119 (4%), respectively (odds ratio (OR) 15.8, *P* < 0.001). Seventeen (5%) of the 363 patients had detectable antibodies against SARS-CoV-2. SARS-CoV-2 seroconversion was detected in 9 (64%) of the RT-PCR-positive patients (Table S1).

Three hundred and 49patients did not undergo any RT-PCR test or had a negative RT-PCR test (Table S2). Eight (2%) of these patients developed antibodies against SARS-CoV-2. Five of these eight had undergone an RT-PCR test, but none experienced COVID-19 symptoms.

SARS-CoV-2-positive patients diagnosed either by RT-PCR or serological test (*n* = 22) did not statistically differ from SARS-CoV-2-negative patients (*n* = 341) with regards to potential risk factors for severe COVID-19 disease (age, comorbidities, smoking, lung cancer, advanced disease, number of lines of systemic therapies for advanced disease, Eastern Cooperative Oncology Group (ECOG) performance status) (Table 1) [20]. However, the SARS-CoV-2-negative patients had received overall more immunotherapy, generating an imbalance between the treatment groups (*P* = 0.029). In terms of cancer types, head and neck cancers and skin cancers were only observed in the SARS-CoV-2 negative group. SARS-CoV-2-positive patients received more thoracic radiotherapy over the previous six months (*P* = 0.007). These same trends were also seen numerically in the COVID-19 subgroups detected through RT-PCR alone (Table S1) or serology alone (Table S2).

### COVID-19 symptoms and thoracic imaging

Among the 363 patients, 150 (41%) developed symptoms suggestive of COVID-19 infection and 213 (59%) remained asymptomatic. Although present in only 5 (23%) SARS-CoV-2-positive patients, anosmia appeared to be the most discriminant symptom, as no anosmia was reported in the SARS-CoV-2-negative patients (*P* < 0.001). Dyspnea and rhinitis were not discriminant, being frequently reported in treated cancer patients. SARS-CoV-2-positive patients presented at least one of the more specific COVID-19 symptoms (fever or cough or anosmia) more frequently during the follow-up period (46% vs 13%, *P* < 0.001) (Table 1). Similar findings were observed in the subgroups of COVID-19 detected through RT-PCR alone (Table S1) or serology alone (Table S2).

Overall, 280 (77%) patients underwent a thoracic computed tomography (CT) scan or a 2′-deoxy-2′-[18F] fluoro-D-glucose positron emission tomography (18-FDG-PET) coupled with a thoracic CT scan during the follow-up period, representing 64 and 78% of the SARS-CoV-2-positive and -negative patients, respectively (*P* = 0.123) (Table 1). These images were pre-planned to evaluate treatment efficacy in 97% of patients. Radiologic signs potentially related to COVID-19 [21] were more frequent in the SARS-CoV-2-positive patients (37% versus 7%, *P* < 0.001). Analyses of the subgroups of patients diagnosed by RT-PCR alone or by serology alone revealed the same trend (Tables S1 and S2). Eight (89%) out of nine patients with a positive RT-PCR with subsequent seroconversion presented COVID-19 symptoms and/or suggestive CT scan findings. In contrast, only one (20%) out of five patients with a positive RT-PCR but a negative serological test presented symptoms and/or suggestive CT scan findings (*P* = 0.023).

### Adverse events reported during systemic oncological treatments

SARS-CoV-2-positive patients identified either by RT-PCR or serology (*n* = 22) presented more hematological adverse events compared to SARS-CoV-2-negative patients (*n* = 341) (73% vs 35%, *P* < 0.001). Adverse events for neutropenia and lymphopenia were of all grades (Table 2). Only grade 1–2 thrombopenia was observed. There were no significant differences between the SARS-CoV-2-positive and -negative patients with regards to other observed adverse events. Bleeding, infections unrelated to COVID-19, and febrile neutropenia occurred at a low rate in the SARS-CoV-2 positive patients (Table 2). Both subgroups of COVID-19 detected only through RT-PCR (Table S3) or only through serology (Table S4) presented similar results. In both univariate and multivariate analyses, SARS-CoV-2 positivity, a lower performance status (ECOG 2–3), and treatment with chemotherapy were significantly associated with hematological toxicity. Advanced disease, higher age, the presence of a comorbidity, and symptoms of COVID-19, were not (Table 3). Table S5 depicts the landscape of treatment regimens the patients received and the number of grade 3–4 hematological adverse events, according to the SARS-CoV-2 status.

**Table 2:** Adverse events recorded during systemic oncological treatment

	All (*N* = 363)	SARS-CoV-2 positive* (*N* = 22)	SARS-CoV-2 negative ^$^ (*N* = 341)	***P***
**Any toxicity**	295 (81%)	19 (83%)	276 (81%)	0.778
**Hematological toxicity**	135 (37%)	16 (73%)	119 (35%)	**< 0.001**
Grade 1–2	78 (22%)	10 (46%)	68 (20%)	
Grade 3–4	56 (16%)	6 (27%)	50 (15%)	
Neutropenia				
Grade 1–2	27 (7%)	5 (23%)	22 (7%)	
Grade 3–4	32 (9%)	4 (18%)	28 (8%)	
Febrile neutropenia	7 (2%)	1 (5%)	6 (2%)	
Lymphopenia				
Grade 1–2	49 (14%)	6 (27%)	43 (13%)	
Grade 3–4	28 (8%)	4 (18%)	24 (7%)	
Thrombopenia				
Grade 1–2	46 (13%)	5 (23%)	41 (12%)	
Grade 3–4	3 (1%)	0	3 (1%)	
Bleeding event	0	0	0	
**Biological toxicity**	117 (32%)	10 (45%)	107 (31%)	0.238
Grade 1–2	107 (30%)	9 (41%)	98 (29%)	
Grade 3–4	9 (3%)	1 (4%)	8 (3%)	
ALT or AST increased				
Grade 1–2	52 (14%)	4 (18%)	48 (14%)	
Grade 3–4	3 (1%)	0	3 (1%)	
alkaline phosphatase increased				
Grade 1–2	61 (17%)	3 (14%)	58 (17%)	
Grade 3–4	4 (1%)	0	4 (1%)	
Blood bilirubin increased				
Grade 1–2	9 (3%)	0	9 (3%)	
Grade 3–4	2 (1%)	0	2 (1%)	
Creatinine increased				
Grade 1–2	23 (6%)	2 (9%)	21 (6%)	
Grade 3–4	3 (1%)	1 (5%)	2 (1%)	
**General adverse events**	235 (65%)	12 (55%)	223 (65%)	0.358
Grade 1–2	227 (63%)	10 (46%)	217 (64%)	
Grade 3–4	8 (2%)	2 (9%)	6 (2%)	
Fatigue				
Grade 1–2	194 (53%)	10 (46%)	184 (54%)	
Grade 3–4	2 (1%)	0	2 (1%)	
Peripheral neuropathy				
Grade 1–2	101 (28%)	5 (23%)	96 (28%)	
Grade 3–4	3 (1%)	2 (9%)	1 (0.3%)	
Rash (acneiform or maculo-papular)				
Grade 1–2	71 (120%)	4 (18%)	67 (20%)	
**Digestive tract toxicity**	143 (39%)	10 (46%)	133 (39%)	0.654
Grade 1–2	139 (38%)	9 (41%)	130 (38%)	
Grade 3–4	4 (1%)	1 (5%)	3 (1%)	
Nausea				
Grade 1–2	83 (23%)	6 (27%)	77 (23%)	
Grade 3–4	1 (0.3%)	1 (5%)	0	
Vomiting				
Grade 1–2	19 (5%)	2 (9%)	17 (5%)	
Grade 3–4	1 (0.3%)	1 (5%)	0	
Diarrhea				
Grade 1–2	90 (25%)	6 (27%)	84 (25%)	
Grade 3–4	4 (1%)	1 (5%)	3 (1%)	
**Infection** ^**α**^	14 (4%)	1 (5%)	13 (4%)	
Airway tract	4 (1%)	0	4 (1%)	
**Anti-cancer treatment delay**	79 (22%)	12 (55%)	67 (20%)	**< 0.001**
Duration				
median (days)	14	14	11	
Interquartile range (days)	7–15	7–21	7–15	0.504

* Defined as patients with SARS-CoV-2 detected by serology or RT-PCR. ^$^ Defined as patients with a negative serology test and either a negative RT-PCR or no RT-PCR performed

^α^ Of any type other than COVID-19. *ALT* alanine aminotransferase, *AST* aspartate transaminase; COVID-19: The viral pandemic coronavirus disease 2019; *SARS-CoV-2* severe acute respiratory syndrome coronavirus-2; Adverse events graded according to Common Terminology Criteria for Adverse Events version 5.0

**Table 3:** Factors potentially associated with hematological toxicity

Factor	Univariate analysis		Multivariate analysis	
	Odds ratio (95% CI)	***P***	Odds ratio (95% CI)	***P***
Age (≥ 65 vs < 65 years)	0.947 (0.617–1.455)	0.804		
SARS-CoV-2 status (positive vs negative)	4.974 (1.897–13.048)	**0.001**	4.061 (1.421–11.606)	**0.009**
Comorbidity (absent vs present)	0.848 (0.552–1.300)	0.449		
Cancer type (other vs lung)	1.260 (0.695–2.284)	0.447		
Disease extent (localized vs metastatic)	1.521 (0.973–2.378)	0.066		
ECOG (2–3 vs 0–1)	5.357 (1.424–20.149)	**0.013**	6.718 (1.517–29.745)	**0.012**
COVID-19 symptoms*: yes vs no	1.192 (0.661–2.151)	0.559		
Treatment:				
Chemotherapy vs immunotherapy	12.172 (5.950–24.900)	**< 0.001**	11.435 (5.519–23.692)	**< 0.001**
Chemotherapy + immunotherapy vs immunotherapy	11.280 (3.898–32.645)	**< 0.001**	10.361 (3.495–30.719)	**< 0.001**
Other vs immunotherapy	2.452 (0.987–6.094)	0.053	2.157 (0.846–5.496)	0.107

* Fever, cough or anosmia

*CI* confidence interval; COVID-19: The viral pandemic coronavirus disease 2019; SARS-CoV-2: severe acute respiratory syndrome coronavirus-2, *ECOG* Eastern Cooperative Oncology Group; vs: versus

SARS-CoV-2-positive patients experienced more frequent treatment delays than SARS-CoV-2-negative patients: 55% vs 20%, respectively (*P* < 0.001). However, the length of delay did not differ between the two groups: median 14 vs 11 days, respectively (*P* = 0.504). The relationship between treatment delays and hematological adverse events was significant (*P* < 0.001, Fisher exact test).

Two SARS-CoV-2-positive patients, detected by RT-PCR and later confirmed by serology, prematurely ended their ongoing systemic treatment during the study period; one developed complicated sigmoid diverticulitis, while the second developed grade 2 peripheral neuropathy. Three SARS-CoV-2-negative patients also stopped treatment prematurely; two due to adverse events and one to disease progression. No patients stopped treatment due to the pandemic.

### Factors associated with seroconversion

To further assess the factors associated with positive SARS-CoV-2 serology, univariate and multivariate analyses were performed (Table 4). SARS-CoV-2 positivity by RT-PCR was the strongest factor associated with seropositivity. The main COVID-related symptoms (fever, cough or anosmia), or a thoracic CT scan with lung infiltrates suggestive of COVID-19 infection, were also significantly associated with seroconversion in both the univariate and multivariate analyses. A higher age, the presence of a comorbidity, lung cancer, recent morbid surgery, or advanced disease were not associated with seroconversion. Recent thoracic radiotherapy was significantly associated with positive serology in the univariate analysis but not in the multivariate analysis.

**Table 4:** Logistic regression of factors potentially associated with positive SARS-CoV-2 serology

Factor	Univariate analysis		Multivariate analysis	
	Odds ratio (95% CI)	***P***	Odds ratio (95% CI)	***P***
Age (≥ 65 vs < 65 years)	0.688 (0.249–1.903)	0.471		
Comorbidity (absent vs present)	1.323 (0.499–3.510)	0.574		
Cancer type (other vs lung)	0.882 (0.245–3.172)	0.848		
Disease extent (localized vs metastatic)	2.350 (0.883–6.254)	0.087		
Recent morbid surgery (yes vs no)	0.622 (0.130–2.973)	0.552		
Thoracic radiotherapy < 6 months ago (yes vs no)	3.588 (1.196–10.767)	**0.023**		
SARS-CoV-2 RT-PCR:				
not performed vs negative	0.334 (0.079–1.423)	0.138	0.422 (0.092–1.941)	0.268
positive vs negative	43.920 (10.697–180.3)	**< 0.001**	31.159 (6.029–161.04)	**< 0.001**
Thoracic CT scan:				
not done vs normal	6.390 (1.561–26.154)	**0.010**	4.698 (0.906–24.362)	0.065
COVID-19 suspected vs normal	28.522 (7.076–114.97)	**< 0.001**	14.574 (2.858–74.313)	**0.001**
COVID-19 symptoms*: yes vs no	7.526 (2.761–20.509)	**< 0.001**	4.080 (1.074–15.502)	**0.039**
Treatment				
Chemotherapy vs others	1.317 (0.359–4.840)	0.678		
Chemotherapy + immunotherapy vs others	0.873 (0.086–8.869)	0.909		
Immunotherapy vs others	0.178 (0.018–1.752)	0.139		

Performance status could not be added to the model as no patient with Eastern Cooperative Oncology Group 2–3 tested positive. Recent thoracic radiotherapy was not retained by the backward stepwise selection in the multivariate modeling

* Fever, cough or anosmia

^α^ Targeted therapy or antibody drug conjugate, approved or as part of a clinical study

*CI* confidence interval, *CT* computed tomography, COVID-19: The viral pandemic coronavirus disease 2019, *SARS-CoV-2* severe acute respiratory syndrome coronavirus-2; vs: versus

### COVID-19 outcomes

Five of the 22 (23%) SARS-CoV-2-positive patients were hospitalized due to COVID-19 infection. Of these, three required transient nasal oxygen therapy. All developed antibodies against SARS-CoV-2. Four additional patients were hospitalized with presumed COVID-19, but their status was validated neither by RT-PCR nor by thoracic imaging. These four patients eventually had negative serology.

### COVID-19 in Belgium, at CUSL, and in the health care workers of the CUSL ambulatory cancer care unit

Table 5 summarizes the COVID-19 pandemic epidemiology in Belgium and the main actions our cancer center implemented according to national and institutional recommendations. Belgian official COVID-19 data are continuously updated on https://covid-19.sciensano.be. Between February 15 and July 13, 2020, only two (7%) out of the 28 physicians who attended the outpatient cancer center daily developed a symptomatic COVID-19 infection documented by SARS-CoV-2 RT-PCR. None of the other physicians nor the unit’s 19 oncological nurses developed a symptomatic infection. All healthcare workers were tested for SARS-CoV-2 antibodies during the same period as the ONCOSARS-1 study. Only the two physicians who were positive for SARS-CoV-2 by RT-PCR developed antibodies against SARS-CoV-2.

**Table 5:** General information regarding COVID-19 epidemiology and timing of the main measures in Belgium and at Cliniques universitaires Saint-Luc (CUSL)

COVID-19 epidemiology in Belgium	
February 3, 2020	1st proven case (repatriation from Wuhan, China)
February 28, 2020	1st proven case in a Belgian resident
April 6, 2020	Peak of the epidemic (6012 patients hospitalized)
July 1, 2020	Results of the sero-epidemiology study from Antwerp Hospital: antibodies against SARS-CoV-2 detected in 5.5% of the population, based on 2960 samples (Van Damme P et al., media communication, July 1, 2020)
**COVID-19 epidemiology in CUSL**	
February 18, 2020	1st case hospitalized
April 2, 2020	Peak of hospitalizations (*n* = 156, 26 in ICU)
**COVID-19 management in CUSL**	
March 2, 2020	RT-PCR test available in-house for symptomatic patients requiring hospitalization
March 14, 2020	Test indicated for healthcare workers with respiratory symptoms and fever without geographic context
March 14, 2020	National hospital emergency plan directing general preventive measures
March 30, 2020	Test indicated for healthcare workers with mild respiratory symptoms
April 1, 2020	Systematic RT-PCR test before each invasive procedure or admission of a hospitalized patient. Due to logistics, systematic RT-PCR was never recommended for ambulatory oncology patients admitted to the day unit.
April 7, 2020	Face masks were made mandatory inside the hospital
**Measures specific to the Department of Medical Oncology at CUSL**	
March 6, 2020	A. Start of systematic check for COVID-19 symptoms: 1) the day before planned systemic treatment (in-house pre-treatment consultation on a different floor to the ambulatory care unit or by phone) 2) a second check was established the day of scheduled treatment at the entrance to the outpatient medical oncology day unit. 3) in case of any symptoms suggestive of COVID-19 infection: - 7 days treatment postponement without any COVID-19 RT-PCR if this treatment delay was judged medically acceptable. - COVID-19 RT-PCR if it was judged that a treatment delay could impact the patient prognosis or if hospitalization was required. B. Exclusion of any visitors to the oncology wards. C. Tissue face masks highly recommended to each patient entering CUSL.

National epidemiology data is based on the daily Sciensano epidemiology bulletin available on https://covid-19.sciensano.be. *CUSL* Cliniques universitaires Saint-Luc, *ICU* intensive care unit; RT-PCR: reverse-transcriptase polymerase chain reaction; COVID-19: The viral pandemic coronavirus disease 2019

## Discussion

The rapid spread and non-negligible risk of a fatal outcome due to COVID-19 has triggered scientific societies and healthcare systems to define strategies to mitigate the risk of infection in vulnerable patients [3, 4]. Here we present the results of a cohort study of ambulatory cancer patients treated with systemic therapy during the first peak of the pandemic period in Belgium.

In our study, only 6% of patients (a finding similar to what is seen at the time of writing this article in the general Belgian population, see Table 5), and 7% of healthcare workers had laboratory proven contact with the virus (positive RT-PCR or detectable anti-SARS-CoV-2 antibodies). We hypothesize that the rapid implementation of preventative measures (systematic screening of patients with fever or acute airway tract symptoms before entering the oncology day unit, prohibiting visitors unless medically advised, the strong recommendation to wear a face mask, and social distancing) was a key factor, at least in part, in our low rate of SARS-CoV-2 infection [22].

Our SARS-CoV-2-positive patients presented more frequently with fever, cough or anosmia. Although clearly associated with COVID-19 in the general population, [20] dyspnea, a frequent symptom of cancer patients, as well as fatigue and diarrhea, frequently reported as treatment-related adverse events, appeared less discriminant in our cancer patients (Tables 1 and 3). This illustrates just one aspect of the diagnostic challenge during this pandemic. We observed that five out of eight (63%) patients who were not diagnosed with COVID-19 during the pandemic, but for which a seroconversion was detected afterwards, had presented at least one COVID-19 related symptom. This highlights the need to implement large COVID-19 screening programs in cancer patients to avoid spread of the virus, even among patients with no or very mild COVID-19 symptoms. We found that SARS-CoV-2-positive patients had more characteristic lung infiltrates on CT-scan. This finding was also observed in SARS-CoV-2-positive patients who were not detected during the three first months of the pandemic but who were subsequently diagnosed through serological testing. This suggests that thoracic imaging is also a useful screening tool.

Delivering optimal systemic anti-cancer treatment while protecting our patients and medical staff are key objectives during this pandemic. Except the implementation of specific protective measures depicted in Table 5, we did not modify our standard day unit treatment protocols. The safety data we report here are therefore reassuring. We only found a higher rate of hematological adverse events in the SARS-CoV-2-positive patients including all grades of neutropenia and lymphopenia, with the latter being the most commonly found biological abnormality in this disease [20]. These two adverse events could thus reflect a COVID-19 symptom without additional treatment toxicity. Hematotoxicity led to a higher rate of treatment delays. Importantly, we did not encounter any increase in the other assessed adverse events in SARS-CoV-2-positive patients, whereas bleeding, infections unrelated to COVID-19, and febrile neutropenia rates were low. A multivariate analysis confirmed that hematological toxicity was significantly associated with the presence of SARS-CoV-2 antibodies after adjusting for patient’s performance status and type of systemic treatment. A poorer performance status and the administration of chemotherapy were also significantly associated with hematological toxicity. Recently published data is consistent with our findings: in a cohort study of 309 COVID-19-positive cancer patients, the administration of chemotherapy was not associated with a severe form of the disease [23]. Likewise, in a cohort study of 1016 cancer patients, SARS-CoV-2 infection rates remained as low as in the general population after the implementation of institutional safety measures [24]. Our patients received a vast landscape of chemotherapy regimens, which prevents us from estimating whether certain specific treatment schedules are more likely to be associated with toxicity.

Previous work hypothesized that cancer patients are less likely to develop or maintain antibodies against SARS-CoV-2 [25]. This study adds data to this assumption, as only nine out of 14 (64%) patients with a positive SARS-CoV-2 RT-PCR test had detectable antibody levels at study inclusion. In this population, the presence of symptoms or lung infiltrates on CT-scan suggestive of COVID-19 was significantly associated with the development of antibodies. However, we could not find definitive risk factors for the development of antibodies against SARS-CoV-2. Administration of radiotherapy to the thoracic area within the previous 6 m was significantly associated with seroconversion in the univariate but not multivariate analysis, warranting exploration in larger cohorts.

Our study has several limitations. First, we prospectively recruited only patients who were still being actively treated with anti-cancer therapy after the first peak of the pandemic. Therefore, we cannot exclude that we missed some SARS-CoV-2-negative and non-severe SARS-CoV-2-positive patients treated during the pandemic who had already stopped treatment before the study began. Second, we cannot guarantee that some medical decisions, such as complementary tests and/or treatment options, were not taken with the pandemic in mind. Third, at the beginning of the pandemic, the decision whether to perform SARS-CoV-2 RT-PCR testing was taken in the context of testing kit shortages. Likewise, it was logistically not feasible to compensate for the lack of tests by offering thoracic CT scans. Fourth, this study focused on solid cancer patients. While haematological cancer patients do not appear at increased risk of SARS-CoV-2 infection, they are at higher risk of developing severe COVID-19 [26]. Our results should thus not be extrapolated to them. Finally, the rate of false negative results from the nasopharyngeal RT-PCR test is reported to be in the order of 30–40% [27]. This reduces the accuracy of any data ever reported for COVID-19. Likewise, several tests exist to detect antibodies against SARS-CoV-2, with highly variable diagnostic accuracies [28]. However, the electrochemiluminescent immunoassay we used has a high sensitivity (91%) and specificity (100%) at the cut-off pre-specified by the manufacturer [29].

## Conclusion

This cohort study of patients with solid cancers actively treated during the COVID-19 pandemic demonstrates that when strong preventive measures are taken to protect patients and healthcare workers, systemic anti-cancer therapy can be safely administered to SARS-CoV-2-positive patients not presenting with a severe form of the infection. Attention should especially focus on hematological adverse events. Typical COVID-19 symptoms (fever, cough or anosmia) should trigger SARS-CoV-2 RT-PCR testing to confirm or rule out SARS-CoV-2 infection.

## Supplementary Information

**Additional file 1: Table S1**. Characteristics of patients who had SARS-CoV-2 RT-PCR. **Table S2**. Characteristics of patients diagnosed by SARS-CoV-2 serology (SARS-CoV-2 RT-PCR not performed or negative). **Table S3**. Adverse events with systemic therapy in the population with RT-PCR results. **Table S4**. Adverse events with systemic therapy in the population without or with negative RT-PCR results. **Table S5**. Landscape of treatment regimens the patients received and the number of grade 3–4 hematological adverse events, according to the SARS-CoV-2 status. **Supplementary appendix 1**: Framework used to notify adverse events at each cycle of anti-cancer treatment.

## Data Availability

All data generated or analyzed during this study are included in this published article and its supplementary information files.

## Abbreviations

18-FDG-PET: 2′-deoxy-2′-[18F] fluoro-D-glucose positron emission tomography
CT: Computed tomography
CTCAE: Common Terminology Criteria for Adverse Events
CUSL: Cliniques universitaires Saint-Luc
ECLIA: Electrochemiluminescent immunoassay
ECOG: **Eastern Cooperative Oncology Group**
RT-PCR: Reverse transcription polymerase chain reaction
SARS-CoV-2: Severe acute respiratory syndrome coronavirus-2

## References

[1] Wiersinga WJ, Rhodes A, Cheng AC, Peacock SJ, Prescott HC (2020). Pathophysiology, transmission, diagnosis, and treatment of coronavirus disease 2019 (COVID-19): a review. JAMA..

[2] Triggle CR, Bansal D, Farag EABA, Ding H, Sultan AA (2020). COVID-19: Learning from Lessons To Guide Treatment and Prevention Interventions. mSphere.

[3] Pelin C, Timothy K, Alison F, Asmita M, Lawrence S, James B (2020). Safety at the time of the COVID-19 pandemic: how to keep our oncology patients and healthcare workers safe. J Natl Compr Canc Netw.

[4] Curigliano G, Banerjee S, Cervantes A, Garassino M, Garrido P, Girard N (2020). Managing cancer patients during the COVID-19 pandemic: an ESMO interdisciplinary expert consensus. Ann Oncol.

[5] ASCO special report : a guide to cancer care delivery during the COVID-19 pandemic. https://www.asco.org/sites/newwww.asco.org/files/content-files/2020-ASCO-Guide-Cancer-COVID19.pdf. Accessed 3 Nov 2020.

[6] Rogado J, Obispo B, Gullón P, Lara M (2021). Impact of the COVID-19 pandemic in cancer diagnosis in the first and second waves in one of the most affected cancer areas in the city of Madrid (Spain). Int J Cancer.

[7] Liang W, Guan W, Chen R, Wang W, Li J, Xu K, Li C, Ai Q, Lu W, Liang H, Li S, He J (2020). Cancer patients in SARS-CoV-2 infection: a nationwide analysis in China. Lancet Oncol..

[8] Yu J, Ouyang W, Chua MLK, Xie C (2020). SARS-CoV-2 transmission in patients with Cancer at a tertiary Care Hospital in Wuhan, China. JAMA Oncol.

[9] Miyashita H, Mikami T, Chopra N, Yamada T, Chernyavsky S, Rizk D (2020). Do patients with cancer have a poorer prognosis of COVID-19? An experience in New York City. Ann Oncol.

[10] Tian Y, Qiu X, Wang C, Zhao J, Jiang X, Niu W (2020). Cancer associates with risk and severe events of COVID-19: a systematic review and meta-analysis. Int J Cancer.

[11] Giannakoulis VG, Papoutsi E, Siempos II (2020). Effect of Cancer on clinical outcomes of patients with COVID-19: a meta-analysis of patient data. JCO Global Oncology.

[12] Saini KS, Tagliamento M, Lambertini M, McNally R, Romano M, Leone M, Curigliano G, de Azambuja E (2020). Mortality in patients with cancer and coronavirus disease 2019: a systematic review and pooled analysis of 52 studies. Eur J Cancer.

[13] de Joode K, Dumoulin DW, Tol J, Westgeest HM, Beerepoot LV, van den Berkmortel F (2020). Dutch oncology COVID-19 consortium: outcome of COVID-19 in patients with cancer in a nationwide cohort study. Eur J Cancer.

[14] Garassino MC, Whisenant JG, Huang LC, Trama A, Torri V, Agustoni F, Baena J, Banna G, Berardi R, Bettini AC, Bria E, Brighenti M, Cadranel J, de Toma A, Chini C, Cortellini A, Felip E, Finocchiaro G, Garrido P, Genova C, Giusti R, Gregorc V, Grossi F, Grosso F, Intagliata S, la Verde N, Liu SV, Mazieres J, Mercadante E, Michielin O, Minuti G, Moro-Sibilot D, Pasello G, Passaro A, Scotti V, Solli P, Stroppa E, Tiseo M, Viscardi G, Voltolini L, Wu YL, Zai S, Pancaldi V, Dingemans AM, van Meerbeeck J, Barlesi F, Wakelee H, Peters S, Horn L, TERAVOLT investigators (2020). COVID-19 in patients with thoracic malignancies (TERAVOLT): first results of an international, registry-based, cohort study. Lancet Oncol.

[15] Hempel D, Milani V, Kleespies A, Hempel L, Ebner F, Zehn D, Keim S (2020). 1680P SARS-CoV-2 infections in outpatients with cancer: Most infected patients are asymptomatic carriers without impact on chemotherapy. Ann Oncol.

[16] Wise-Draper TM, Desai A, Elkrief A, Rini BI, Flora DB, Bowles DW, Shah D, Rivera D, Johnson DB, Lopes G, Grivas P, Thompson MA, Peters S, Kuderer NM, Nock NL, Grover P, Li X, Gulati S, Choueiri TK, Warner J (2020). LBA71 systemic cancer treatment-related outcomes in patients with SARS-CoV-2 infection: a CCC19 registry analysis. Ann Oncol.

[17] Roche Diagnostics. Elecsys Anti-SARS-CoV-2, insert sheet REF 09203079190. 202007, V 1.0. https://diagnostics.roche.com/content/dam/diagnostics/Blueprint/en/pdf/cps/Elecsys-Anti-SARS-CoV-2-factsheet-2020-JUL.pdf. Accessed 3 Nov 2020.

[18] Harris PA, Taylor R, Thielke R, Payne J, Gonzalez N, Conde JG (2009). Research electronic data capture (REDCap)--a metadata-driven methodology and workflow process for providing translational research informatics support. J Biomed Inform.

[19] Harris PA, Taylor R, Minor BL, Elliott V, Fernandez M, O'Neal L (2019). The REDCap consortium: Building an international community of software platform partners. J Biomed Inform.

[20] Guan W-J, Ni Z-Y, Hu Y, Liang W-H, Ou C-Q, He J-X (2020). Clinical characteristics of coronavirus disease 2019 in China. N Engl J Med.

[21] Simpson S, Kay FU, Abbara S, Bhalla S, Chung JH, Chung M, Henry TS, Kanne JP, Kligerman S, Ko JP, Litt H (2020). Radiological society of North America expert consensus statement on reporting chest CT findings related to COVID-19. Endorsed by the Society of Thoracic Radiology, the American College of Radiology, and RSNA - secondary publication. J Thorac Imaging.

[22] Wang X, Ferro EG, Zhou G, Hashimoto D, Bhatt DL (2020). Association between universal masking in a health care system and SARS-CoV-2 positivity among health care workers. JAMA..

[23] Jee J, Foote MB, Lumish M, Stonestrom AJ, Wills B, Narendra V, et al. Chemotherapy and COVID-19 Outcomes in Patients With Cancer. J Clin Oncol. 2020:Jco2001307. 10.1200/JCO.20.01307 PMC7571792 32795225

[24] Berghoff AS, Gansterer M, Bathke AC, Trutschnig W, Hungerländer P, Berger JM, et al. SARS-CoV-2 Testing in Patients With Cancer Treated at a Tertiary Care Hospital During the COVID-19 Pandemic. J Clin Oncol. 2020:Jco2001442. 10.1200/JCO.20.01442 PMC7571795 32795227

[25] Solodky ML, Galvez C, Russias B, Detourbet P, N'Guyen-Bonin V, Herr AL (2020). Lower detection rates of SARS-COV2 antibodies in cancer patients versus health care workers after symptomatic COVID-19. Ann Oncol.

[26] Sahu KK, Cerny J (2020). Managing patients with hematological malignancies during COVID-19 pandemic. Expert Rev Hematol.

[27] Wang W, Xu Y, Gao R, Lu R, Han K, Wu G, Tan W (2020). Detection of SARS-CoV-2 in different types of clinical specimens. JAMA..

[28] Lisboa Bastos M, Tavaziva G, Abidi SK, Campbell JR, Haraoui LP, Johnston JC (2020). Diagnostic accuracy of serological tests for covid-19: systematic review and meta-analysis. BMJ (Clin Res Ed).

[29] Favresse J, Eucher C, Elsen M, Tré-Hardy M, Dogné JM, Douxfils J (2020). Clinical performance of the Elecsys Electrochemiluminescent immunoassay for the detection of SARS-CoV-2 Total antibodies. Clin Chem.

