# Supplementary material for: Safety of systemic anti-cancer treatment in oncology patients with non-severe COVID-19: a cohort study
Source: BMC Cancer. 2021 May 20;21:578. doi: 10.1186/s12885-021-08349-8 (PMC8134961; doi:10.1186/s12885-021-08349-8)
Supplement: Supplementary file 1 — Additional file 1: Table S1. Characteristics of patients who had SARS-CoV-2 RT-PCR. Table S2. Characteristics of patients diagnosed by SARS-CoV-2 serology (SARS-CoV-2 RT-PCR not performed or negative). Table S3. Adverse events with systemic therapy in the population with RT-PCR results. Table S4. Adverse events with systemic therapy in the population without or with negative RT-PCR results. Table S5. Landscape of treatment regimens the patients received and the number of grade 3–4 hematological adverse events, according to the SARS-CoV-2 status. Supplementary appendix 1: Framework used to notify adverse events at each cycle of anti-cancer treatment. [file 12885_2021_8349_MOESM1_ESM.docx]

**TABLE S1. Characteristics of patients who had SARS-CoV-2 RT-PCR**

|  | **All**  (*N*=141) | **RT-PCR positive**  (*N*=14) | **RT-PCR  negative**  (*N*=127) |
| --- | --- | --- | --- |
| RT-PCR test   - Positive - Negative | 14 (10%)  127 (90%) | 14 (100%)  0 | 0  127 (100%) |
| Serological test   - Positive - Negative | 14 (10%)  127 (90%) | 9 (64%)  5 (36%) | 5 (4%)  122 (96%) |
| Age   - Median (IQR) - ≥ 65 years old | 61 (53-70)  57 (40%) | 58 (43-68)  5 (38%) | 62 (54-70)  52 (41%) |
| Gender   - Male - Female | 64 (45%)  77 (55%) | 8 (57%)  6 (43%) | 56 (44%)  71 (56%) |
| Comorbidities   - Arterial hypertension - Diabetes - Cirrhosis - CKD - COPD - Heart disease - Auto-immune disease | 78 (55%)  48 (34%)  17 (12%)  2 (1%)  12 (9%)  9 (6%)  16 (11%)  8 (6%) | 9 (64%)  5 (36%)  1 (7%)  0  3 (21%)  1 (7%)  3 (21%)  1 (7%) | 69 (54%)  43 (34%)  16 (13%)  2 (2%)  9 (7%)  8 (6%)  13 (10%)  7 6%) |
| Cancer type   - Breast/gynecological - Genito-urinary - Lung - Digestive tract - Skin - Head and neck - Sarcoma - Other ^α^ | 42 (30%)  12 (9%)  25 (18%)  44 (31%)  6 (4%)  2 (1%)  5 (4%)  5 (4%) | 4 (29%)  1 (7%)  4 (29%)  3 (21%)  0  0  0  2 (14%) | 38 (30%)  11 (9%)  21 (17%)  41 (32%)  6 (5%)  2 (2%)  5 (4%)  3 (2%) |
| Cancer stage   - Loco-regional - Metastatic   - Lung   - Other visceral   - Non-visceral | 59 (42%)  82 (58%)  31 (22%)  29 (21%)  22 (16%) | 7 (50%)  7 (50%)  2 (14%)  4 (29%)  1 (7%) | 52 (41%)  75 (59%)  29 (23%)  25 (20%)  21 (17%) |
| ECOG   - 0-1 - 2-3 | 134 (95%)  7 (5%) | 14 (100%)  0 | 120 (95%)  7 (6%) |
| Cancer treatment   - Chemotherapy - Immunotherapy - Chemotherapy + immunotherapy - Other ^β^ - Line of treatment if metastatic (mean ± SD) | 90 (64%)  25 (18%)  13 (9%)  13 (9%)  2 ± 1.2 | 10 (72%)  1 (7%)  2 (14%)  1 (7%)  1 ± 1.9 | 80 (63%)  24 (19%)  11 (9%)  12 (9%)  2 ± 1.2 |
| Other factors   - Smoker - Thoracic radiotherapy < 6 months ago - Heavy surgery < 6 months ago | 66 (47%)  20 (14%)  25 (18%) | 6 (43%)  4 (29%)  1 (7%) | 60 (47%)  16 (13%)  24 (19%) |
| COVID-19 symptoms   - Any - Fever, cough or anosmia - Fever - Cough - Anosmia - Rhinitis - dyspnea | 71 (50%)  32 (23%)  14 (10%)  23 (16%)  3 (2%)  15 (11%)  57 (40%) | 7 (50%)  6 (43%)  3 (21%)  5 (36%)  1 (7%)  2 (14%)  6 (43%) | 64 (50%)  26 (21%)  11 (9%)  18 (14%)  2 (2%)  13 (10%)  51 (40%) |
| Thoracic imaging   - Performed   - COVID-19 suspected | 112 (79%)  17 (12%) | 9 (64%)  5 (36%) | 103 (81%)  12 (10%) |

^α^ Chordoma, multiple primary tumor, brain tumors.

^β^ Targeted therapy or antibody drug conjugate, approved or as part of a clinical study.

SARS-CoV-2: severe acute respiratory syndrome coronavirus-2; ECOG: Eastern Cooperative Oncology Group; COPD: chronic obstructive pulmonary disease; CKD: chronic kidney disease; RT-PCR: reverse-transcriptase polymerase chain reaction; SD: standard deviation. COVID-19: The viral pandemic coronavirus disease 2019; NA: non-applicable

**TABLE S2. Characteristics of patients diagnosed by SARS-CoV-2 serology (SARS-CoV-2 RT-PCR not performed or negative)**

|  | **All**  (*N*=349) | **serology positive**  (*N*=8) | **serology  negative**  (*N*=341) |
| --- | --- | --- | --- |
| RT-PCR test   - Positive - Negative | 127 (36%)  0  127 (36%) | 5 (63%)  0  5 (63%) | 122 (36%)  0  122 (36%) |
| Serological test   - Positive - Negative | 8 (2%)  341 (98%) | 8 (100%)  0 | 0  341 (100%) |
| Age   - Median (IQR) - ≥ 65 years old | 63 (55-71)  154 (44%) | 52 (43-66)  3 (38%) | 63 (56-71)  151 (44%) |
| Gender   - Male - Female | 159 (46%)  190 (54%) | 4 (50%)  4 (50%) | 155 (46%)  186 (55%) |
| Comorbidities   - Arterial hypertension - Diabetes - Cirrhosis - CKD - COPD - Heart disease - Auto-immune disease | 186 (53%)  120 (34%)  42 (12%)  6 (2%)  28 (8%)  18 (5%)  40 (12%)  22 (6%) | 3 (38%)  2 (25%)  1 (13%)  0  0  0  1 (13%)  1 (13%) | 183 (54%)  118 (35%)  41 (12%)  6 (2%)  28 (8%)  18 (5%)  39 (11%)  21 (6%) |
| Cancer type   - Breast/gynecological - Genito-urinary - Lung - Digestive tract - Skin - Head and neck - Sarcoma - Other ^α^ | 107 (31%)  26 (8%)  54 (16%)  90 (26%)  30 (9%)  18 (5%)  12 (3%)  12 (3%) | 2 (25%)  0  0  4 (50%)  0  0  1 (13%)  1 (13%) | 105 (31%)  26 (8%)  54 (16%)  86 (25%)  30 (9%)  18 (5%)  11 (3%)  11 (3%) |
| Cancer stage   - Loco-regional - Metastatic   - Lung   - Other visceral   - Non-visceral | 114 (33%)  235 (67%)  86 (25%)  82 (24%)  67 (19%) | 3 (38%)  5 (63%)  3 (38%)  1 (13%)  1 (13%) | 111 (33%)  230 (67%)  83 (24%)  81 (24%)  66 (19%) |
| ECOG   - 0-1 - 2-3 | 337 (97%)  12 (3%) | 8 (100%)  0 | 329 (97%)  12 (4%) |
| Cancer treatment   - Chemotherapy - Immunotherapy - Chemotherapy + Immunotherapy - Other ^β^ - Line of treatment if metastatic (mean ± SD) | 169 (48%)  103 (30%)  20 (6%)  57 (16%)  2 ± 1.3 | 5 (63%)  0  0  3 (38%)  2 ± 0.6 | 164 (48%)  103 (30%)  20 (6%)  54 (16%)  2 ± 1.4 |
| Other factors   - Smoker - Thoracic radiotherapy < 6 months ago - Heavy surgery < 6 months ago | 160 (46%)  37 (11%)  43 (12%) | 3 (38%)  3 (38%)  2 (25%) | 157 (46%)  34 (10%)  41 (12%) |
| COVID-19 symptoms   - Any - Fever, cough or anosmia - Fever - Cough - Anosmia - Rhinitis - Dyspnea | 143 (41%)  48 (14%)  15 (4%)  38 (11%)  4 (1%)  28 (8%)  119 (34%) | 5 (63%)  4 (50%)  2 (25%)  1 (13%)  4 (50%)  2 (25%)  4 (50%) | 138 (41%)  44 (13%)  13 (4%)  37 (11%)  0  26 (8%)  115 (34%) |
| Thoracic imaging   - Performed   - COVI-19 suspected | 271 (78%)  26 (8%) | 5 (63%)  3 (38%) | 266 (78%)  23 (7%) |

^α^ Eye melanoma, chordoma, multiple primary tumor, thymic carcinoma, brain tumors.

^β^ Targeted therapy or antibody drug conjugate, approved or as part of a clinical study.

SARS-CoV-2: severe acute respiratory syndrome coronavirus-2; ECOG: Eastern Cooperative Oncology Group; COPD: chronic obstructive pulmonary disease; CKD: chronic kidney disease; RT-PCR: reverse-transcriptase polymerase chain reaction; SD: standard deviation. COVID-19: The viral pandemic coronavirus disease 2019; NA: non-applicable

**TABLE S3. Adverse events with systemic therapy in the population with RT-PCR results**

|  | **All**  (*N*=141) | **RT-PCR positive**  (*N*=14) | **RT-PCR  negative**  (*N*=127) | ***P*** |
| --- | --- | --- | --- | --- |
| **Any toxicity** | 122 (87%) | 13 (93%) | 109 (86%) | 0.693 |
| **Haematological toxicity**   - - Grade 1-2   - Grade 3-4 | 75 (53%)  42 (30%)  33 (23%) | 12 (86%)  7 (50%)  5 (36%) | 63 (50%)  35 (28%)  28 (22%) | **0.011** |
| Neutropenia   - - Grade 1-2   - Grade 3-4   - Febrile neutropenia | 15 (11%)  21 (15%)  6 (4%) | 4 (29%)  3 (21%)  1 (7%) | 11 (9%)  18 (14%)  5 (4%) |  |
| Lymphopenia   - - Grade 1-2   - Grade 3-4 | 28 (20%)  16 (11%) | 4 (29%)  4 (29%) | 24 (19%)  12 (9%) |  |
| Thrombopenia   - - Grade 1-2   - Grade 3-4 | 28 (20%)  2 (1%) | 3 (21%)  0 | 25 (20%)  2 (2%) |  |
| **Biological toxicity**   - - Grade 1-2   - Grade 3-4 | 59 (42%)  54 (38%)  5 (4%) | 7 (50%)  6 (43%)  1 (7%) | 52 (41%)  48 (38%)  4 (3%) | 0.575 |
| ALT or AST increased   - - Grade 1-2   - Grade 3-4 | 27 (19%)  2 (1%) | 2 (14%)  0 | 25 (20%)  2 (2%) |  |
| alkaline phosphatase increased   - - Grade 1-2   - Grade 3-4 | 31 (22%)  3 (2%) | 2 (14%)  0 | 29 (23%)  3 (2%) |  |
| Blood bilirubin increased   - - Grade 1-2   - Grade 3-4 | 5 (4%)  1 (1%) | 0  0 | 5 (4%)  1 (1%) |  |
| Creatinine increased   - - Grade 1-2   - Grade 3-4 | 10 (7%)  1 (1%) | 2 (14%)  1 (7%) | 8 (6%)  0 |  |
| **General adverse events**   - - Grade 1-2   - Grade 3-4 | 95 (67%)  91 (64%)  4 (3%) | 8 (57%)  7 (50%)  1 (7%) | 87 (69%)  84 (66%)  3 (2%) | 0.385 |
| Fatigue   - - Grade 1-2   - Grade 3-4 | 79 (56%)  1 (1%) | 7 (50%)  0 | 72 (57%)  1 (1%) |  |
| Peripheral neuropathy   - - Grade 1-2   - Grade 3-4 | 38 (27%)  3 (2%) | 3 (21%)  1 (7%) | 35 (28%)  2 (2%) |  |
| Rash (acneiform or maculo-papular)   - - Grade 1-2 | 20 (14%) | 2 (14%) | 18 (14%) |  |
| **Digestive tract toxicity**   - - Grade 1-2   - Grade 3-4 | 67 (48%)  65 (46%)  2 (1%) | 7 (50%)  6 (43%)  1 (7%) | 60 (47%)  59 (47%)  1 (1%) | 0.999 |
| Nausea   - - Grade 1-2   - Grade 3-4 | 39 (28%)  1 (1%) | 4 (29%)  1 (7%) | 35 (28%)  0 |  |
| Vomiting   - - Grade 1-2   - Grade 3-4 | 10 (7%)  1 (1%) | 1 (7%)  1 (7%) | 9 (7%)  0 |  |
| Diarrhea   - - Grade 1-2   - Grade 3-4 | 45 (31%)  2 (1%) | 5 (36%)  1 (7%) | 40 (32%)  1 (1%) |  |
| **Infection ^α^**  Airway tract | 7 (5%)  1 (1%) | 1 (7%)  0 | 6 (5%)  1 (1%) |  |
| **Anti-cancer treatment delay**  Duration   - - median (days)   - Interquartile range (days) | 46 (33%)  13  7 – 15 | 9 (64%)  14  5 – 14 | 37 (29%)  13  7 – 15 | **0.008** |

**^α^** Of any type other than COVID-19.

ALT: alanine aminotransferase; AST: aspartate transaminase; COVID-19: The viral pandemic coronavirus disease 2019; SARS-CoV-2: severe acute respiratory syndrome coronavirus-2; RT-PCR: reverse-transcriptase polymerase chain reaction.

Adverse events graded according to Common Terminology Criteria for Adverse Events version 5.0

**TABLE S4. Adverse events with systemic therapy in the population without or with negative RT-PCR results**

|  | **All**  (*N*=349) | **serology positive**  (*N*=8) | **serology  negative**  (*N*=341) | **P-value** |
| --- | --- | --- | --- | --- |
| **Any toxicity** | 282 (81%) | 6 (75%) | 276 (81%) | 0.653 |
| **Haematological toxicity**   - - Grade 1-2   - Grade 3-4 | 122 (35%)  71 (20%)  51 (15%) | 4 (50%)  3 (38%)  1 (13%) | 118 (35%)  68 (20%)  50 (15%) | 0.460 |
| Neutropenia   - - Grade 1-2   - Grade 3-4   - Febrile neutropenia | 23 (7%)  29 (8%)  6 (2%) | 1 (13%)  1 (13%)  0 | 22 (7%)  28 (8%)  6 (2%) |  |
| Lymphopenia   - - Grade 1-2   - Grade 3-4 | 45 (13%)  24 (7%) | 2 (25%)  0 | 43 (13%)  24 (7%) |  |
| Thrombopenia   - - Grade 1-2   - Grade 3-4 | 43 (12%)  3 (1%) | 2 (25%)  0 | 41 (12%)  3 (1%) |  |
| **Biological toxicity**   - - Grade 1-2   - Grade 3-4 | 109 (31%)  101 (29%)  8 (2%) | 3 (38%)  3 (38%)  0 | 106 (31%)  98 (29%)  8 (2%) | 0.711 |
| ALT or AST increased   - - Grade 1-2   - Grade 3-4 | 50 (14%)  3 (1%) | 2 (25%)  0 | 48 (14%)  3 (1%) |  |
| alkaline phosphatase increased   - - Grade 1-2   - Grade 3-4 | 59 (17%)  4 (1%) | 1 (13%)  0 | 58 (17%)  4 (1%) |  |
| Blood bilirubin increased   - - Grade 1-2   - Grade 3-4 | 9 (3%)  2 (1%) | 0  0 | 9 (3%)  2 (1%) |  |
| Creatinine increased   - - Grade 1-2   - Grade 3-4 | 21 (6%)  2 (1%) | 0  0 | 21 (6%)  2 (1%) |  |
| **General adverse events**   - - Grade 1-2   - Grade 3-4 | 227 (65%)  220 (63%)  7 (2%) | 4 (50%)  3 (38%)  1 (13%) | 223 (65%)  217 (64%)  6 (2%) | 0.458 |
| Fatigue   - - Grade 1-2   - Grade 3-4 | 187 (54%)  2 (1%) | 3 (38%)  0 | 184 (54%)  2 (1%) |  |
| Peripheral neuropathy   - - Grade 1-2   - Grade 3-4 | 98 (28%)  2 (1%) | 2 (25%)  1 (13%) | 96 (28%)  1 (0%) |  |
| Rash (acneiform or maculo-papular)   - - Grade 1-2 | 69 (20%) | 2 (25%) | 67 (20%) |  |
| **Digestive tract toxicity**   - - Grade 1-2   - Grade 3-4 | 136 (39%)  133 (38%)  3 (1%) | 3 (38%)  3 (38%)  0 | 133 (39%)  130 (38%)  3 (1%) | 0.999 |
| Nausea   - - Grade 1-2   - Grade 3-4 | 79 (23%)  0 | 2 (25%)  0 | 77 (23%)  0 |  |
| Vomiting   - - Grade 1-2   - Grade 3-4 | 18 (5%)  0 | 1 (13%)  0 | 17 (5%)  0 |  |
| Diarrhea   - - Grade 1-2   - Grade 3-4 | 85 (24%)  3 (1%) | 1 (13%)  0 | 84 (25%)  3 (1%) |  |
| **Infection ^α^**  Airway tract | 13 (4%)  4 (1%) | 0  0 | 13 (4%)  4 (1%) |  |
| **Anti-cancer treatment delay**  Duration   - - median (days)   - Interquartile range (days) | 70 (20%)  14  7 – 15 | 3 (38%)  21  17 – 26 | 67 (20%)  11  7 – 15 | 0.233 |

**^α^** Of any type other than COVID-19.

ALT: alanine aminotransferase; AST: aspartate transaminase; COVID-19: The viral pandemic coronavirus disease 2019; SARS-CoV-2: severe acute respiratory syndrome coronavirus-2; RT-PCR: reverse-transcriptase polymerase chain reaction.

Adverse events graded according to Common Terminology Criteria for Adverse Events version 5.0.

**TABLE S5. Landscape of treatment regimens the patients received and the number of grade 3-4 hematological adverse events, according to the SARS-CoV-2 status**

|  | **SARS-CoV-2 positive** | | **SARS-CoV2-negative** | |
| --- | --- | --- | --- | --- |
|  | **n** | **grade 3-4 hematological toxicity** | **n** | **grade 3-4 hematological toxicity** |
| **Breast/gynecological** | 6 | 3 | 105 | 19 |
| doxorubicin-cyclophosphamide - paclitaxel | 2 | 2 | 14 | 2 |
| doxorubicin-cyclophosphamide - paclitaxel + anti-HER2 | 1 | 1 | 17 | 3 |
| maintenance anti-HER2 | 3 | 0 | 25 | 1 |
| carboplatin-paclitaxel | 0 | 0 | 9 | 3 |
| 5FU | 0 | 0 | 1 | 0 |
| capecitabine | 0 | 0 | 1 | 1 |
| capecitabine + anti-HER2 | 0 | 0 | 1 | 0 |
| Carboplatin | 0 | 0 | 1 | 1 |
| PARP inhibitor | 0 | 0 | 1 | 0 |
| Carboplatin-gemcitabine | 0 | 0 | 3 | 2 |
| Navelbine | 0 | 0 | 1 | 1 |
| eribuline | 0 | 0 | 5 | 2 |
| eribuline + anti-HER2 | 0 | 0 | 3 | 1 |
| Nab-paclitaxel + relacolirant | 0 | 0 | 3 | 0 |
| anti-PD1 | 0 | 0 | 6 | 0 |
| anti-CD25 antibody | 0 | 0 | 2 | 0 |
| trastuzumab emtansine | 0 | 0 | 12 | 2 |
| **Genito-urinary** | 1 | 0 | 26 | 3 |
| bleomycin-etoposid-cisplatin | 1 | 0 | 0 | 0 |
| Cabazitaxel | 0 | 0 | 3 | 1 |
| docetaxel | 0 | 0 | 1 | 1 |
| platinum salt - gemcitabine | 0 | 0 | 2 | 0 |
| enfortumab vedotin | 0 | 0 | 1 | 0 |
| anti-PD1 | 0 | 0 | 12 | 0 |
| anti-PD1 + anti-VEGF | 0 | 0 | 3 | 0 |
| anti-PDL1 + PARP inhibitor | 0 | 0 | 1 | 0 |
| anti-PSMA + anti-CD3 | 0 | 0 | 1 | 1 |
| anti-VEGF | 0 | 0 | 1 | 0 |
| anti-androgen | 0 | 0 | 1 | 0 |
| **Lung** | 4 | 1 | 54 | 8 |
| Pemetrexed + anti-PD1 | 1 | 0 | 1 | 0 |
| Docetaxel + anti-VEGF | 1 | 1 | 0 | 0 |
| docetaxel + targeted therapy (other than anti-VEGF) | 0 | 0 | 2 | 0 |
| anti-PD1/PDL1 | 1 | 0 | 28 | 1 |
| antiCTLA4 | 0 | 0 | 1 | 0 |
| carboplatin-paclitaxel + anti-PD1/PDL1 | 0 | 0 | 3 | 1 |
| carboplatin-paclitaxel + anti-VEGF + anti-PDL1 | 1 | 0 | 1 | 0 |
| platinum salt-pemetrexed | 0 | 0 | 2 | 1 |
| cisplatin + etoposide | 0 | 0 | 2 | 2 |
| platinum salt-etoposide + anti-PD1 | 0 | 0 | 3 | 1 |
| cisplatin + navelbine | 0 | 0 | 3 | 0 |
| navelbine | 0 | 0 | 1 | 1 |
| Pemetrexed | 0 | 0 | 2 | 0 |
| Pemetrexed + anti-PDL1 | 0 | 0 | 1 | 0 |
| platinum salt-pemetrexed + anti-PD1 | 0 | 0 | 3 | 1 |
| trastuzumab emtansine | 0 | 0 | 1 | 0 |
| **Digestive tract** | 7 | 2 | 86 | 16 |
| 5FU-bevacizumab | 0 | 0 | 2 | 1 |
| 5FU-atezolizumab | 0 | 0 | 1 | 1 |
| 5FU-trastuzumab | 0 | 0 | 1 | 0 |
| 5FU-nal-irinotecan | 0 | 0 | 1 | 0 |
| 5FU-panitumumab | 0 | 0 | 2 | 0 |
| Capecitabine | 0 | 0 | 1 | 0 |
| Capecitabine-bevacizumab | 1 | 0 | 2 | 0 |
| Capecitabine- nivolumab | 0 | 0 | 1 | 0 |
| Capecitabine-oxaliplatin | 0 | 0 | 5 | 0 |
| Cisplatin-5FU-trastuzumab | 1 | 0 | 0 | 0 |
| Cisplatin-Gemcitabine | 0 | 0 | 5 | 0 |
| Folfiri | 1 | 1 | 6 | 2 |
| Folfiri+cetuximab/panitumumab | 0 | 0 | 4 | 1 |
| Folfiri+bevacizumab | 1 | 0 | 3 | 1 |
| Folfirinox+bevacizumab | 0 | 0 | 6 | 1 |
| Folfox | 2 | 0 | 12 | 2 |
| folfox-bevacizumab | 0 | 0 | 1 | 0 |
| folfox-cetuximab/panitumumab | 0 | 0 | 2 | 0 |
| Gemcitabine | 1 | 1 | 2 | 0 |
| Gemcitabine-nab-paclitaxel | 0 | 0 | 8 | 3 |
| gemcitabine-oxaliplatin | 0 | 0 | 1 | 0 |
| irinotecan-bevacizumab | 0 | 0 | 1 | 0 |
| irinotecan-avelumab-cetuximab | 0 | 0 | 1 | 0 |
| trifluridine-tipiracil + GM102 | 0 | 0 | 5 | 4 |
| paclitaxel + ramucirumab | 0 | 0 | 1 | 0 |
| paclitaxel | 0 | 0 | 2 | 0 |
| anti-PD1 | 0 | 0 | 6 | 0 |
| panitumumab | 0 | 0 | 1 | 0 |
| cetuximab-encorafenib-cobimetinib | 0 | 0 | 3 | 0 |
| **Skin** | 0 | 0 | 30 | 1 |
| Anti-PD-1 | 0 | 0 | 28 | 1 |
| IMCgp100 | 0 | 0 | 2 | 0 |
| **Head and neck** | 0 | 0 | 18 | 1 |
| paclitaxel | 0 | 0 | 1 | 1 |
| Cisplatin-5FU-Pembrolizumab | 0 | 0 | 1 | 0 |
| Anti-PD1/PDL1 | 0 | 0 | 12 | 0 |
| cetuximab | 0 | 0 | 4 | 0 |
| **Sarcoma** | 1 | 0 | 11 | 1 |
| Doxorubicine | 1 | 0 | 3 | 1 |
| Doxorubicine - Dacarbazine | 0 | 0 | 2 | 0 |
| Adriamycine - Trabectedin | 0 | 0 | 1 | 0 |
| Trabectedin | 0 | 0 | 4 | 0 |
| paclitaxel | 0 | 0 | 1 | 0 |
| **Other *** | 3 | 0 | 11 | 1 |
| paclitaxel | 0 | 0 | 1 | 0 |
| folfox | 0 | 0 | 1 | 1 |
| Carboplatin-paclitaxel | 0 | 0 | 1 | 0 |
| Temozolomide | 1 | 0 | 0 | 0 |
| carboplatin-pemetrexed | 0 | 0 | 1 | 0 |
| anti-PD1 | 0 | 0 | 4 | 0 |
| IMCgp100 | 0 | 0 | 2 | 0 |
| Dabrafenib + Trametinib | 1 | 0 | 0 | 0 |
| EOS884448 (anti-TIGIT) | 1 | 0 | 1 | 0 |

* Eye melanoma, chordoma, multiple primary tumor, thymic carcinoma, brain tumors.

**SUPPLEMENTARY APPENDIX 1**:
Framework used to notify adverse events at each cycle of anti-cancer treatment

Pre-treatment consultation : **DATE – PATIENT NAME**

**Diagnosis :**

**Therapy :**

- Regimen:
- Number of planned cycles:
- Cycle n°:
- Date of cycle:

**COVID-19 symptoms since February 2020 ?**

|  | **Yes** | **No** | **Comment** |
| --- | --- | --- | --- |
| Cough |  |  |  |
| Rhinitis |  |  |  |
| Fever |  |  |  |
| Anosmia |  |  |  |
| Other |  |  |  |

**Anamnesis :**

|  | **Yes** | **No** | **Comment** | |
| --- | --- | --- | --- | --- |
| Pain |  |  | Location: | Intensity: /10 |
| Pain killers |  |  | Efficacy: | |
| Fever |  |  |  | |
| Use of antibiotics |  |  |  | |

|  | **Yes** | **No** | **G I** | **G II** | **G III** | **G IV** | **Linked to  treatment** | **Linked to disease** | **Comment** |
| --- | --- | --- | --- | --- | --- | --- | --- | --- | --- |
| Fatigue |  |  |  |  |  |  |  |  |  |
| Nausea |  |  |  |  |  |  |  |  |  |
| Vomiting |  |  |  |  |  |  |  |  |  |
| Diarrhea |  |  |  |  |  |  |  |  |  |
| Constipation |  |  |  |  |  |  |  |  |  |
| Mucositis |  |  |  |  |  |  |  |  |  |
| Anorexia |  |  |  |  |  |  |  |  |  |
| Dyspnea |  |  |  |  |  |  |  |  |  |
| Abnormal heart pulse |  |  |  |  |  |  |  |  |  |
| Edema |  |  |  |  |  |  |  |  |  |
| Hand foot syndrome |  |  |  |  |  |  |  |  |  |
| Lacrimation |  |  |  |  |  |  |  |  |  |
| Sensitive polyneuropathy |  |  |  |  |  |  |  |  |  |
| Motor polyneuropathy |  |  |  |  |  |  |  |  |  |
| Skin symptom |  |  |  |  |  |  |  |  |  |
| Other |  |  |  |  |  |  |  |  |  |
